# BONE RECONSTRUCTION IN THE TREATMENT OF TIBIAL HEMIMELIA: AN ALTERNATIVE TO AMPUTATION?

**DOI:** 10.1590/1413-785220243201e268462

**Published:** 2024-05-06

**Authors:** Yesmin Naji Sola, Luna Jeannie Alves Mangueira, Pedro Miranda Portugal, Robson Xavier Ferro, Nayme Naji Sola, Gustavo Teixeira Leão

**Affiliations:** 1. Hospital Estadual de Urgencias Governador Otavio Lage de Siqueira, Goiania, GO, Brazil.; 2. Hospital Municipal Tide Setubal, Sao Miguel, SP, Brazil.; 3. Universidade Federal de Goias, Hospital das Clínicas, Goiania, GO, Brazil.

**Keywords:** Tibial Hemimelia, Reconstruction, Amputation, Outcomes, Tibia Hemimelial, Reconstrução, Amputação, Medidas de Desfecho

## Abstract

**Objective:**

To evaluate the advantages and disadvantages of bone reconstruction and lengthening compared to amputation in the treatment of tibial hemimelia for patients and their families.

**Methods:**

Systematic review of articles published in English and Portuguese between 1982 and 2022 in the MEDLINE, PubMed, Cochrane and SciELO databases. The variables of interest were: year of publication, sample characteristics, classification of tibial hemimelia according to Jones, treatment outcome and follow-up time.

**Results:**

A total of eleven articles were included in the scope of this review. The studies involved 131 patients, 53.4% male and 46.6% female. The age of the patients who underwent a surgical procedure ranged from 1 year and 10 months to 15 years. The most common type was Jones’ I (40.9%). The most recurrent complications in the reconstruction treatment were: infection of the external fixator path, flexion contracture (mainly of the knee), reduction in the range of motion of the knee and ankle.

**Conclusion:**

We did not find enough relevant studies in the literature to prove the superiority of reconstruction. Amputation remains the gold standard treatment for tibial hemimelia to this day. ***Level of Evidence III, systematic review of level III studies***

## RESUMO

**Objetivo:**

*Avaliar as vantagens e desvantagens da reconstrução óssea e alongamento comparada à amputação no tratamento da hemimelia tibial para pacientes e familiares.*

**Métodos:**

*Revisão sistemática, com análise de artigos publicados nas línguas inglesa e portuguesa entre 1982 e 2022, nas bases de dados MEDLINE, PubMed, Cochrane e SciELO. As variáveis de interesse foram: ano de publicação, característica da amostra, classificação da hemimelia tibial segundo Jones, desfecho do tratamento e tempo de seguimento.*

**Resultados:**

*Fizeram parte do escopo desta revisão onze artigos. Os estudos envolveram 131 pacientes, 53,4% do sexo masculino e 46,6% do feminino. A idade dos pacientes submetidos a algum procedimento cirúrgico variou de 1 ano e 10 meses a 15 anos. O tipo mais comum foi o I de Jones (40,9%). As complicações mais recorrentes no tratamento pela reconstrução foram: infecção do trajeto de pinos do fixador externo, contratura em flexão (principalmente do joelho), redução do arco de movimento de joelho e tornozelo.*

**Conclusão:**

*Não encontramos na literatura estudos suficientemente relevantes para comprovar a superioridade da reconstrução. A amputação se mantém até os dias de hoje o tratamento padrão-ouro para hemimelia tibial.* ***Nível de evidência III; revisão sistemática de estudos de nível III.***

## INTRODUCTION

 Tibial hemimelia is a rare deformity of the lower limbs affecting one in every 1,000,000 live births. ^1^ It ranges from hypoplasia of the tibia to its total absence. The fibula is usually present, and may be dysplastic. ^1^ This disease occurs unilaterally or bilaterally, with an estimated 30% bilaterality, associated with syndromes or other deformities. ^2^

 Clinically, the individual may have a flexed or unstable knee, with absence of central and peripheral ligaments, fixed equinus varus deformity of the foot, polydactyly and medial ray deficiency. The spectrum of presentation of this pathology is much broader when compared to fibular hemimelia. ^2^

 Several associated deformities in the upper limbs are also found, such as: radius dysplasia, lobster claw deformity, syndactyly, triphalangism. ^3^ To date, in the literature, there is no specific genetic mutation identified as a cause of this pathology. ^4^

 In 1978, Jones, Barnes and Lloyd-Roberts ^5^ proposed a classification for tibial hemimelia which is still the most currently widely used. It divides the disability into four main groups, based on radiographs and skeletal morphology. Type 1 (a, b) consists of an absent tibia with or without a normal femoral epiphysis. Type 2 consists of an intact fibula with the presence of a proximal tibia and an absent distal tibial segment. Type 3 is a rare variety with an intact fibula and absent proximal tibia, and present distal tibial. Type 4 is distal tibiofibular diastasis. ^6^

 The treatment of this comorbidity remains controversial. The initial literature proposed amputation and possible prosthetization as the treatment method of choice for the most severe types, Jones’ 1A and 1B. Studies supporting this option showed satisfactory results, especially if performed early. ^7^

 As medical propaedeutics advanced, orthopedic surgeons have been increasingly trying to use procedures that preserve the limb and its functionality. In 1965, Brown ^8^ described a technique based on centralizing the fibula below the femur with the aim of “tibializing” the used bone. Due to the multiple flexion contractures and limb discrepancy, the bone reconstruction was associated with lengthening by using external fixators, mainly according to the Ilizarov technique. Correction is then carried out gradually, with tissue distraction and adequate functionality of the lower limb joints involved. In 2016, Paley ^3^ modified the technique. Despite its growing use, its benefits and success are still uncertain, especially in terms of remaining contractures, prolonged treatment time and associated complications. As a result, there is still no suitable protocol for the surgical treatment of tibial hemimelia. 

The aim of this study was to determine, through a systematic review, the advantages and disadvantages of bone reconstruction and lengthening for patients and their families compared to amputation, which is considered the gold standard in the treatment of tibial hemimelia.

## MATERIALS AND METHODS

 The formulation of the question and search strategy of the article were based on the PICO model (Population, Intervention Comparison, Outcome), widely used in evidence-based practice methodology and recommended for the construction of systematic reviews. The PRISMA model was used as a reference for the article selection flowchart. ^9^

### Search strategy

Articles published from 1980 to 2022 in English and Portuguese describing the treatment of tibial hemimelia and its outcomes, whether amputation or reconstruction were searched in the following databases: MEDLINE, PUBMED, COCHRANE and SCIELO. The initial search used the descriptors hemimelia combined with tibia, lower extremity deformities, and congenital.

Articles were selected by two independent examiners on the basis of reading the title and abstract. Potentially eligible articles were read in full. The examiners then checked the reference lists of all eligible articles attempting to find new references for this review.

### Elegibility criteria

The inclusion criteria were: (1) population (adults or children); (2) intervention (bone reconstruction and lengthening or amputation); (3) outcome (functionality, quality of life); (4) articles published in the last 42 years – in English and Portuguese; (5) reviews with meta-analysis, clinical trials, cohort studies, case series, clinical cases; (6) studies with full text available in the searched databases.

### Data extraction

After carrying out the previous steps, a reviewer proceeded to extract the following data from each article: year of publication, sample characteristics (sample size, population, age, gender), classification of tibial hemimelia treated according to Jones, outcome of the applied treatment and follow-up time.

The variables of interest were transferred by one of the authors to an Excel spreadsheet (Microsoft Corp., United States). The data of interest was treated using descriptive statistics. Due to the great heterogeneity of the studies, it was not possible to conduct a meta-analysis.

## RESULTS

Based on the used descriptors and the date of publication indicated by the authors, a total of: 90 articles, 17 from PUBMED, 71 from MEDLINE, 2 from SCIELO and none from Cochrane were found.

Considering the eligibility criteria, 70 articles were excluded after reading the title and abstract. Among the most common reasons for exclusion were: studies that did not involve treatment, studies on genetic factors of the disease, concept studies, studies that did not include the outcome of the treatment applied. Articles that did not have full text available were also excluded.

**Figure 1.**
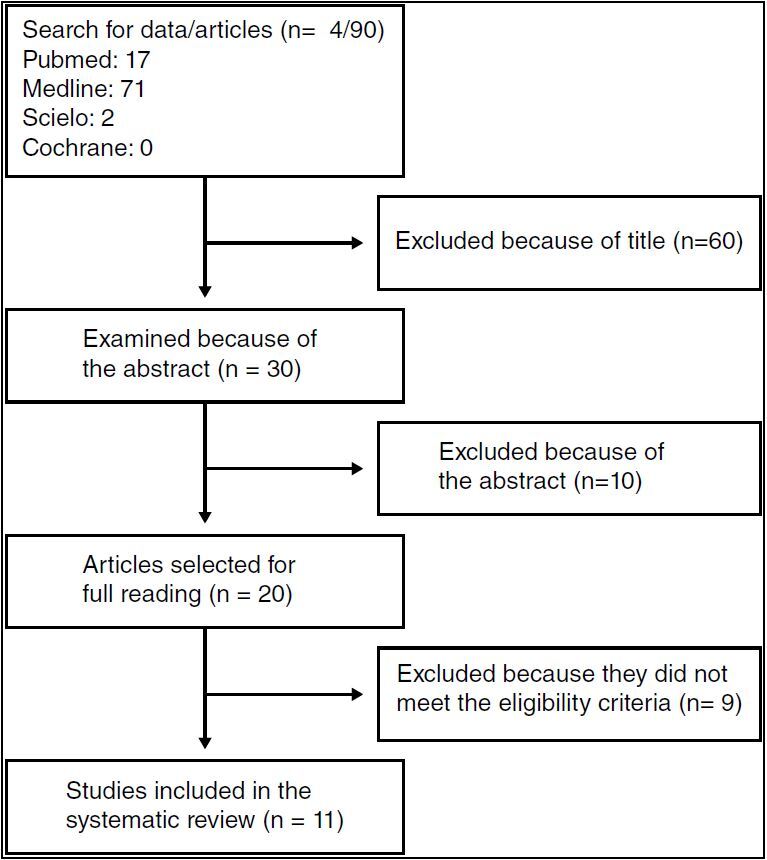
Fluxogram.

 The 20 selected studies were checked for duplication, which found no identical articles. Subsequently, these articles were read in full and nine of them were excluded because they did not present relevant data for the review. After the selection stages and application of eligibility criteria, 11 articles were included in this systematic review. The PRISMA model flowchart was used to illustrate the process ( Figure 1 ). 

 Next, the extracted data was summarized in a table for better visualization with the following variables: year of publication, type of study, sample size and characteristics, classification of tibial hemimelia, patient follow-up time and outcome of the used treatment ( Table 1 ). 

**Table 1.** Analysis of the studies included in the systematic review

**Author/Yenr**	**Type of study**	**Sample size**	**Sex**	**Age**	**Classification**	**Follow-up time**	**Outcomes**
Laufer et al. (2020) ^1^	Retrospective case series study	10 patients (2 with bilaterality)	7 male; 3 female	Mean age: 2,3 years old	2 patients IV B (Paley); 4 patients VA; 6 patients VC	Average follow-up: 7.1 years	Mobility improved in all patients. All were able to walk with a full load and without pain, but all required knee-ankle-foot orthoses. All were able to participate in daily life normally. All relatives said that they had seen a great improvement compared to the preoperative situation and that they would opt for limb salvage treatment again. Despite the findings, the article concluded that amputation still has fewer complications and should be considered the gold standard.
Spiegel et al. (2003) ^10^	Retrospective case series study	15 patients (4 with bilaterality)	10 male; 5 female	Mean age: 1 year and 10 months old	10 type I de Jones; 5 type II; 4 type III	Average follow-up: 7 years	All type I patients were treated with knee disarticulation without post-operative complications. Type II disabilities were treated with foot amputation (Syme or Chopart) and tibiofibular synostosis. No prosthetization problems were identified during follow-up. Type III cases were treated with Syme amputation, and two developed complications, including symptomatic instability in the proximal or distal joint. Regarding reconstructions, there are still no available guidelines to draw firm conclusions.
Balci et al. (2015) ^11^	Retrospective case series study	21 patients (7 with bilaterality)	12 male; 9 female	Mean age: 4.8 years old	7 Jones type IA; 4 type 1B; 11 type II; 1 type III; 5 type IV	Average follow-up: 5.8 years old	All the disarticulated knees (6) were Jones type IA. One patient with type III underwent transtibial amputation. In the other patients, Brown’s method associated with an external fixator was used. There were 14 complications: 3 flexion contractures > 30° in the knee joint, 2 equinus deformities, 3 knee dislocations, 2 knee subluxations and 4 plastic deformities. In Jones type IA cases the SF36 questionnaire was much higher in those who underwent disarticulation than those who underwent reconstruction. The study showed that disarticulation was not superior to reconstruction, except in type IA patients.
Youssef Ahmed (2014) ^12^	Retrospective case series study	8 patients	5 male; 3 female	Mean age: 2.3 years old	8 Jones type II	Average follow-up: 2.6 years	There were positive results in all eight cases, with a good range of motion in the knee and plantigrade foot, and all patients walked and had no pain. All cases showed total patient and parent satisfaction. The article stated that by comparing the results obtained from limb salvage with those of amputations and prosthetic replacements, in terms of functional outcome, duration of treatment, incidence of complications and the number of surgical procedures required, amputation would be much better.
Carraza-Bencano e González-Rodríguez (1999) ^13^	Case report	1 patient	1 female	15 years old	Jones type II	Average follow-up: 2 years	The LC-monotube external fixator was used as a treatment to correct the 13.5 cm discrepancy, with the hindfoot in 60° varus, with the forefoot slightly in adduction and supination. At the end of the follow-up, the patient was walking without the aid of canes, with notable clinical and functional improvement. A plantigrade foot was obtained, with a stable ankle that did not require shoe modifications, allowing the patient to walk and run freely without limitations.
Fernandez-Palazzi, Bendahan e Rivas (1998) ^14^	Retrospective case series study	18 patients (4 with bilaterality)	7 male; 11 female	Mean age: not mentioned	9 Jones type IA cases; 4 type IB; 3 type II; 4 type IV	Average follow-up: not mentioned	In 10 type Ia and Ib cases, knee disarticulation was performed. One type II case underwent below-knee amputation and proximal tibiofibular synostosis. Two type III cases were treated with Syme amputation. Only in the type IV deformity was reconstructed. The article concluded that amputation is the procedure with the lowest cost and best adaptation. The Brown procedure did not meet expectations.
Hosny (2005) ^15^	Retrospective case series study	6 patients	3 male; 3 female	Mean age: 7.5 years old	2 Jones type IA; 4 type II	Average follow-up: 3 years	In type IA cases, Ilizarov was applied from the femur to the foot. The Brown procedure was performed one month later. Families were satisfied in all cases. Infection in the pin tract occurred in all cases, which were treated with oral antibiotics. Knee flexion deformity remained in two cases. Fracture of the femur occurred in one case. It was believed that the method of treating tibial hemimelia described in this series can be appreciated in cases in which amputation is refused, as marked functional improvement can be expected.
Loder e Herring (1987) ^16^	Retrospective case series study	6 patients (3 with bilaterality)	3 male; 3 female	Mean age: 9. 5 months old	Classification not mentioned	Average follow-up: from 1 year and 8 months to 10 years and 3 months	Five out of nine knees were considered to have a good result, with contracture in flexion < 10 degrees, and three achieved full extension. Subsequently, all nine subsequently developed increased flexion contractures. Only one knee maintained active quadriceps strength. Three developed varus and medial subluxation, and one underwent disarticulation. According to the Jakayakumar and Eilert criteria, no limb achieved satisfactory results.
Shrivastava et al. (2009) ^17^	Case report	1 patient	1 male	Age: 9 years old	Not included in Jones’ classification	Follow-up: 4 years	The total lengthening of the fibula achieved during reconstruction was 23 cm. The external fixator was applied for 635 days. The range of movement of the knee was 0-90 (active) and 0-110 (passive). The knee showed no signs of instability. At the end of the follow-up, the patient was able to walk without pain. There were no major complications. The article suggests that amputation can be avoided with proper planning and salvage surgery.
Courvoisier et al. (2009) ^18^	Retrospective case series study	9 patients (1 with bilaterality)	5 male; 4 female	Mean age: 2 years and 1 month old	4 Jones type I; 5 type II	Average follow-up: 18.3 years	The Ilizarov method was used in five cases in combination with the Brown technique. One case evolved with knee disarticulation. One patient progressed to bilateral knee arthrodesis. The average maximum knee flexion was 35 (0-90) in type I deficiencies and 118 (90-140) in type II deficiencies. In two patients, knee stabilization was achieved at the end of the correction. Due to the associated anomalies often reported in type I congenital tibial deficiencies, amputation remained the treatment of choice.
Shahcheraghi e Javid (2016) ^19^	Cohort study	36 patients (12 with bilaterality)	17 male; 19 female	Mean age: 12 years and 1 month old	14 Jones type I, 16 types II, no type III, 11 type IV. 7 of the cases were not classified in any Jones subtype.	Average follow-up: 9 years	Knee movement was normal for all except those with previous joint abnormalities. The ankle was quite stiff in 14 cases and 22 had around 15 degrees of range of motion. Quality of life was assessed using the PedsQL score, indicating 68 points in the reconstruction group and 64.6 in the amputee group. The reconstruction group obtained a better functional score than the amputee group in 4 items: physical, social, psychological and school. Among the amputee group, 4 were totally satisfied and 4 were partially satisfied. In the reconstruction group, 8 were totally satisfied, 19 were almost satisfied and 1 was dissatisfied.

 The studies involved 131 patients, 53.4% of whom were male and 46.6% female. The age of the patients who underwent a surgical procedure ranged from 1 year and 10 months old to 15 years old, but most of them were treated as early as possible. Among the limbs operated on, the most common variant, according to Jones’ classification, was type 1 (40.9%), followed by type 2, (40.1%). However, as a bias, there was one study that did not mention classification, ^16^ another that classified patients according to Paley’s classification, ^1^ a case report ^17^ with a variant of presentation that could not be classified according to Jones and a study with seven patients that also did not fit into the types proposed by Jones. ^19^ Bilaterality was found in 33 patients (25.2% of cases), which is in line with literature information. ^2^

Among the main complications found by the authors regarding the treatment anchored in reconstruction, we can mention: infection of the external fixator pin path, but all patients had satisfactory resolution after using oral antibiotics; maintenance or new flexion contracture, especially of the knee; reduction in the range of movement of the knee and ankle, and the need for multiple surgical procedures. These complications were not found in the group of patients who underwent amputation or disarticulation.

 All articles used Brown’s method, with centralization of the fibula on the femur, as one of the types of employed treatment. However, there was no homogeneity in deciding which type of classification would be used: disarticulation, amputation or salvage surgery, which constituted a bias to the comparative evaluation of the methods. Only one article ^11^ systematized treatment satisfaction using the SF36 instrument; the others assessed satisfaction and post-operative quality of life descriptively, with the main parameter being the individual’s ability to walk without the aid of orthopedic supports. One article ^19^ used the PedsQL questionnaire to assess post-operative quality of life of patients who underwent amputation compared to those that underwent reconstruction. 

## DISCUSSION

 The first reported case of tibial hemimelia was described in 1841, and by 1941 around 79 cases had been published. The diagnosis can be made in the uterus, from the 16th week of pregnancy, by ultrasound. Genetic inheritance varies, with cases of autosomal dominant and autosomal recessive transmission reported. ^2^

 The most widely used classification was proposed by Jones in 1978, which divides the form of presentation of tibial hemimelia into four main groups, the first group being subdivided into two others. ^1^^,^^10^ In 2003, Dror Paley proposed another classification, which was modified in 2015. ^3^ There are five main types and 11 subtypes. Type 1 is the hypoplastic but not deficient tibia, with increased growth of the fibula. Type 2 has a proximal and distal tibial epiphysis, but a dysplastic ankle. Type 3 presents distal tibiofibular diastasis and absence of the tibial pilon. Type 4 is marked by distal tibial aplasia with preservation of the proximal epiphysis. Finally, type 5 corresponds to complete tibial aplasia with the knee in flexion contracture. ^3^

 The importance of these classifications is mainly in terms of treatment and prognosis guided by the subtypes. Initially, treatment was based on amputation or disarticulation at the level of the knee, especially for Jones subtypes Ia and Ib. ^12^ However, this therapeutic proposal is not always welcomed in certain cultures. Kumar Sahoo et al. ^20^ published a cohort study of 24 patients with tibial deficiency in India in 2019. Of these, only one patient opted for amputation treatment, showing that in some countries such acceptance is still low. 

 Treatment based on bone reconstruction and lengthening was instituted for the affected joints, ankle and knee, with the aim of improving stability, function and aesthetics. ^15^ Among the treatments proposed and the studies used here, we can mention: for cases of complete absence of the tibia, centralization of the fibula over the femoral condyles with fusion or arthroplasty (initially described by Brown). ^8^ For cases of partial tibial deficiency, the following options are available: synostosis of the tibial remnant with the fibula, contralateral transposition of the fibula, fusion of the fibula with the talus, transfer of the fibula proximal to the femoral intercondylar notch and both proximal and distal synostosis of the tibia with the fibula. ^3^ The choice of the best reconstruction method varies according to the patient’s profile, the classification of the tibial hemimelia, the surgeon’s experience and the quality of the quadriceps muscles, as well as the degree of flexion in the knee and ankle joints. 

 To date, we have found no studies in the literature with sufficient basis to guide the choice of treatment for each case of tibial hemimelia, whether amputation or reconstruction. The function of the quadriceps with knee extension seems to be mandatory in order to obtain satisfactory results when centralizing the fibula as a treatment. The objectives of the proposed and chosen treatment are: to keep the foot plantigrade, to keep the knee joint functional, to maintain stability of the ankle joint, most of the time using arthrodesis as an instrument, and to maintain adequate limb length quality. ^11^^-^^13^

 In 2015, Shahcheraghi and Javid, ^19^ presented a study with the largest sample of patients with tibial hemimelia undergoing some kind of treatment. Out of a total of 36 patients, 26 were treated with bone reconstruction and eight underwent an amputation procedure. This is one of the few studies comparing patient satisfaction and quality of life using a questionnaire. Quality of life was assessed using the PedsQL score, which showed 68 points in the reconstruction group and 64.6 in the amputee group. The reconstruction group obtained a better functional score than the amputee group in four items: physical, social, psychological and school. In terms of “satisfaction”, the amputee group had four patients who were totally satisfied and four who were partially satisfied. In the reconstruction group, out of the 28 patients, eight were totally satisfied, 19 were almost satisfied and one was dissatisfied. 

 In 2015, Balci et al. ^11^ also presented results regarding the quality of life of patients treated with reconstruction compared to those who opted for amputation. The authors used the SF36 questionnaire and concluded that the results were much higher in patients who underwent amputation. The most common complications of the reconstruction procedures were: knee flexion contractures, knee instability, decreased range of motion of the knee and ankle and the need for multiple serial procedures, infection of the external fixator pin tract, as well as reoperations. 

 Fernandez-Palazzi, Bendahan and Rivas ^14^ performed disarticulation on all the patients they approached with tibial hemimelia classified as Jones type Ia and Ib, with the initial argument that Brown’s procedure (centralization of the fibula) does not show adequate functional results for the limb. Another argument used by authors, who advocate amputation, is based on adaptation. The younger the patient undergoes the procedure, the faster their physiological accommodation and viability to prosthetization. 

 The choice of treatment that includes reconstruction and bone lengthening is still new and depends on the surgeon’s experience. In 2021, Dror Paley published an article indicating his preference for reconstruction. ^2^ The procedure was performed on a sample of 250 patients. In addition to correcting the limb discrepancy, it also corrected equinus varus deformities of the foot. Treatment was gradually conducted, using an external fixator associated with internal synthesis, such as femoral osteotomy and patelloplasty if necessary. Despite describing the technique and presenting a large sample, the author did not evidence the complications related to the procedure. ^2^

Despite the improvement in reconstructive surgery for the treatment of tibial hemimelia over the last decade, there is still not enough data in the literature on long-term results to help devise a treatment protocol, especially in cases of complete tibial agenesis.

## CONCLUSION

The study had some limitations, such as the small sample of patients approached, non-standardization of groups of patients undergoing amputation or reconstruction according to Jones’ classification, and the variety of long-term results and described complications.

After extensive reading and according to the table presented, amputation is still the first choice of treatment, especially for Jones type Ia and Ib cases. This procedure saved patients from multiple approaches and, in most of the cases presented, still brought the best functional outcome and adaptation for them. Reconstruction is a complex, long-term treatment modality with a high rate of complications.

In conclusion, reconstruction surgery can be offered with the combination of osteogenic distraction principles in patients with tibial hemimelia. The patient and their family should be approached, and their treatment’s expectations understood. Distraction osteogenesis treatment techniques and Ilizarov’s principles should be applied by experienced surgeons in specialized centers.

## References

[1] Laufer A., Frommer A., Gosheger G., Roedl R., Broeking J. N., Toporowski G. (2020). Femoro-pedal distraction in staged reconstructive treatment of tibial aplasia. Bone Joint J.

[2] Chong D. Y., Paley D. (2021). Deformity reconstruction surgery for tibial hemimelia. Children.

[3] Paley D. (2016). Tibial hemimelia: new classification and reconstructive options. J Child Orthop.

[4] Litrenta J., Young M., Birch J. G., Oetgen M. E. (2019). Congenital tibial deficiency. J Am Acad Orthop Surg.

[5] Jones D., Barnes J., Lloyd-Roberts G. C. (1978). Congenital aplasia and dysplasia of the tibia with intact fibula: classification and management. J Bone Joint Surg Br.

[6] Senthil V., Kottamttavide Shah, H. (2016). Unclassified tibial hemimelia. BMJ Case Rep.

[7] Farr S., Ganger R., Grill F. (2014). Congenital tibial hemimelia. Orthopade.

[8] Brown F. W. (1965). Construction of a knee joint in congenital total absence of the tibia (paraxial hemimelia tibia): a preliminary report. J Bone Joint Surg Am.

[9] Moher D., Liberati A., Tetzlaff J., Altman D. G., PRISMA Group (2009). Preferred reporting items for systematic reviews and meta-analyses: the PRISMA statement. PLoS Med.

[10] Spiegel D. A., Loder R. T., Crandall R. C. (2003). Congenital longitudinal deficiency of the tibia. Int Orthop.

[11] Balcı H. İ., Sağlam Y., Bilgili F., Şen C., Kocaoğlu M., Eralp L. (2015). Preliminary report on amputation versus reconstruction in treatment of tibial hemimelia. Acta Orthop Traumatol Turc.

[12] Youssef Ahmed A. A. Y. (2014). Staged soft tissue, bony and Ilizarov procedures for correction of leg and foot deformities in tibial hemimelia. Clin Res Foot Ankle.

[13] Carranza-Bencano A., González-Rodríguez E. (1999). Unilateral tibial hemimelia with leg length inequality and varus foot: external fixator treatment. Foot Ankle Int.

[14] Fernandez-Palazzi F., Bendahan J., Rivas S. (1998). Congenital deficiency of the tibia: a report on 22 cases. J Pediatr Orthop B.

[15] Hosny G. A. (2005). Treatment of tibial hemimelia without amputation: preliminary report. J Pediatr Orthop B.

[16] Loder R. T., Herring J. A. (1987). Fibular transfer for congenital absence of the tibia: a reassessment. J Pediatr Orthop.

[17] Shrivastava S., Nawghare S., Dulani R., Singh P., Jain S. (2009). A rare variant of tibial hemimelia and its treatment. J Pediatr Orthop B.

[18] Courvoisier A., Sailhan F., Thevenin-Lemoine C., Vialle R., Damsin J. P. (2009). Congenital tibial deficiencies: treatment using the Ilizarov’s external fixator. Orthop Traumatol Surg Res.

[19] Shahcheraghi G. H., Javid M. (2016). Functional assessment in tibial hemimelia (can we also save the foot in reconstruction?). J Pediatr Orthop.

[20] Kumar Sahoo P., Sahu M. M., Prasad Das S. (2019). Clinical spectrum of congenital tibial hemimelia in 35 limbs of 24 patients: a single center observational study from India. Eur J Med Genet.

